# A genome-wide association study of childhood adiposity and blood lipids

**DOI:** 10.12688/wellcomeopenres.16928.1

**Published:** 2021-11-10

**Authors:** Katie O'Nunain, Eleanor Sanderson, Michael V Holmes, George Davey Smith, Tom G Richardson

**Affiliations:** 1 Bristol Medical School, University of Bristol, Bristol, BS8 2BN, UK; 2 MRC Integrative Epidemiology Unit, University of Bristol, Bristol, BS8 2BN, UK; 3 Medical Research Council Population Health Research Unit, University of Oxford, Oxford, OX3 7LF, UK; 4 Clinical Trial Service Unit & Epidemiological Studies Unit, University of Oxford, Oxford, OX3 7LF, UK; 5 Novo Nordisk Research Centre, Oxford, OX3 7FZ, UK

**Keywords:** Early life adiposity, lipoprotein lipids, cardiometabolic disease, genetic correlations, ALSPAC

## Abstract

**Background:** The rising prevalence of childhood obesity and dyslipidaemia is a major public health concern due to its association with morbidity and mortality in later life.

**Methods:** In this study, we have conducted genome-wide association studies (GWAS) for eight measures of adiposity and lipids in a cohort of young individuals (mean age 9.9) from the Avon Longitudinal Study of Parents and Children (ALSPAC). These measures were body mass index (BMI), systolic and diastolic blood pressure, high-density and low-density lipoprotein cholesterol, triglycerides, apolipoprotein A-I and apolipoprotein B. We next undertook functional enrichment, pathway analyses and linkage disequilibrium (LD) score regression to evaluate genetic correlations with later-life cardiometabolic diseases.

**Results:** Using GWAS we identified 14 unique loci associated with at least one risk factor in this cohort of age 10 individuals (P<5x10 ^-8^), with lipoprotein lipid-associated loci being enriched for liver tissue-derived gene expression and lipid synthesis pathways. LD score regression provided evidence of various genetic correlations, such as childhood systolic blood pressure being genetically correlated with later-life coronary artery disease (rG=0.26, 95% CI=0.07 to 0.46, P=0.009) and hypertension (rG=0.37, 95% CI=0.19 to 0.55, P=6.57x10 ^-5^), as well as childhood BMI with type 2 diabetes (rG=0.35, 95% CI=0.18 to 0.51, P=3.28x10 ^-5^).

**Conclusions:** Our findings suggest that there are genetic variants inherited at birth which begin to exert their effects on cardiometabolic risk factors as early as age 10 in the life course. However, further research is required to assess whether the genetic correlations we have identified are due to direct or indirect effects of childhood adiposity and lipid traits.

## Introduction

Childhood obesity is a growing epidemic estimated to affect over 100 million children globally ( GBD 2015 Obesity Collaborators *et al.*, 2017). Early intervention for this disease is crucial owing to its detrimental influence on children’s psychological and physical health ( Vander Wal & Mitchell, 2011). Furthermore, childhood obesity and dyslipidaemia are associated with an increased risk of cardiovascular disease, type 2 diabetes and hypertension in later life ( Ayer *et al.*, 2015; Baker *et al.*, 2007; Pulgaron & Delamater, 2014). These chronic disease outcomes have a poor prognosis and place a considerable economic burden on healthcare systems worldwide ( Wang *et al.*, 2011). This emphasises the importance of understanding the early life influences of adiposity and lipoprotein lipid traits, even though previous studies have suggested that they ultimately influence cardiometabolic disease outcomes if their levels remain high for many years across the life course ( Bjerregaard & Baker, 2018; Newman *et al.*, 1990; Richardson *et al.*, 2020b).

There is strong evidence of a genetic contribution to adiposity, such as previous studies estimating the heritability of body mass index (BMI) at 40% ( Hemani *et al.*, 2013; Robinson *et al.*, 2017). Although there have been numerous genome-wide association studies (GWAS) to date of childhood BMI ( Bradfield *et al.*, 2019; Felix *et al.*, 2016; Vogelezang *et al.*, 2020), there have been far fewer GWAS of blood pressure ( Parmar *et al.*, 2016), and in particular lipoprotein lipid traits, based on measures during childhood.

In this study, we have conducted GWAS of eight measures of adiposity and lipoprotein lipids within a population of young individuals (mean age 9.9) from the Avon Longitudinal Study of Parents and Children (ALSPAC) ( Boyd *et al.*, 2013). These were BMI, systolic blood pressure (SBP), diastolic blood pressure (DBP), high-density lipoprotein (HDL) cholesterol, low-density lipoprotein (LDL) cholesterol, triglycerides, apolipoprotein A-I and apolipoprotein B. We next undertook functional enrichment analyses to highlight the putative underlying tissue types responsible for our GWAS results and to investigate whether they were overrepresented amongst curated biological pathways. In doing so we sought to recapitulate findings from large-scale studies of adult populations, therefore reinforcing that the genome-wide loci identified in our study begin to exert their effects on traits in childhood. Finally, we conducted linkage disequilibrium (LD) score regression to evaluate genetic correlations of childhood adiposity and blood lipid traits with later-life cardiometabolic disease endpoints.

## Methods

### The Avon Longitudinal Study of Parents and Children (ALSPAC)

ALSPAC is a transgenerational cohort study designed to investigate the influence of genetic and environmental factors on the health of both parents and children. The details of the study are described elsewhere ( Boyd *et al.*, 2013; Fraser *et al.*, 2013). In brief, the study recruited 13,761 pregnant women who lived in South West England and were due to deliver between the 1st April 1991 and 31st December 1992. These women and their children have been followed up at regular intervals over the past 27 years. Detailed phenotypic information, biological samples and genetic data have been collected from the participants which are available through a searchable data dictionary ( http://www.bris.ac.uk/alspac/researchers/our-data/). Written informed consent was obtained for all study participants. Ethical approval for this study was obtained from the ALSPAC Ethics and Law Committee and the Local Research Ethics Committees.

***Genotyping and imputation.*** Genome-wide genotyping was undertaken on ALSPAC offspring at a cohort level with quality control, cleaning and imputation, as described previously ( Boyd *et al.*, 2013). Genotype data on participants was derived using the Illumina HumanHap550 quad genome-wide single nucleotide polymorphism (SNP) genotyping platform (Illumina Inc, San Diego, USA) by the Wellcome Trust Sanger Institute (WTSI, Cambridge, UK) and the Laboratory Corporation of America (LCA, Burlington, NC, USA). Samples were excluded based on the following criteria: incorrect sex assignment; abnormal heterozygosity (<0.320 or >0.345 for WTSI data; <0.310 or >0.330 for LCA data); high missingness (>3%); cryptic relatedness (>10% identity by descent) and non-European ancestry (detected by multidimensional scaling analysis). After conducting quality control (QC), the final directly genotyped dataset contained 526,688 SNP loci.

Genotypes with minor allele frequency > 0.01 and Hardy-Weinberg equilibrium P > 5×10 ^-7^ were firstly phased together using ShapeIt (version 2, revision 727) ( Delaneau *et al.*, 2013), before undertaking imputation using Impute (v2.2.2) ( Howie *et al.*, 2009), with a reference panel from the 1000 Genomes project (phase 1, version 3, phased using ShapeIt version 2, December 2013, using all populations). Subsequently, imputation dosages were converted to best-guess genotypes and filtered to only keep variants with an imputation quality score ≥ 0.8. The final imputed dataset used for the analyses presented here contained 8,074,398 loci.

***Cardiometabolic exposures.*** We selected eight measures of early-life adiposity and blood lipids from the ALSPAC study to analyse in this research. The measurements were taken from participants who attended the ALSPAC clinic at age 9 (mean age 9.9, range 8.8–11.7) and are detailed as follows. BMI was calculated using the equation weight[kg]/height[m ^2^], with weight and height measured to the nearest 0.1kg and 0.1cm, respectively. Systolic blood pressure (SBP) and diastolic blood pressure (DBP) were measured while the participants were at rest using a Dinamap 9301 monitor. Two readings were taken for each, the mean of which was used in our analysis. Plasma lipid concentrations were calculated by taking non-fasting blood samples from the participants. High-density lipoprotein (HDL) cholesterol, total cholesterol and triglycerides were measured by modifying the standard Lipid Research Clinics Protocol with lipid determining reagents ( Cooper *et al.*, 1988). LDL cholesterol was determined using the Friedewald equation ( Friedewald *et al.*, 1972). Apolipoprotein A-I and apolipoprotein B were calculated using immunoturbidimetric assays (Roche).

Before undertaking analyses, cardiometabolic trait data were cleaned to identify outliers and to check distributions for normality. Outliers were removed from the analysis and were defined as any value four standard deviations (SD) greater or less than the mean. We applied log transformations to ensure normality when distributions were skewed. Individuals with withdrawn consent or those that had an older sibling in the dataset were removed. The mean, SD and sample size for each cleaned trait are listed in Supplementary Table 1 ( *Underlying data,* O Nunain *et al.*, 2021a).

### Statistical analysis

***Genome-wide association study in the ALSPAC cohort.*** GWAS were conducted for each trait using PLINK v 2.0 software with adjustment for age and sex ( Chang *et al.*, 2015). Adjustment for population ancestry is vital as population stratification can introduce confounding and produce spurious associations ( Price *et al.*, 2006). Therefore, we repeated analyses for any identified GWAS hits with additional adjustment for the top 10 principal components to verify that our results were not affected by population stratification.

A p-value threshold of 5×10 ^-8^ was used to assess whether any of the associations reached conventional genome-wide significance corrections. An LD clumping cut-off of r ^2^<0.001 was applied to identify independent genetic variants using the 1000 Genomes reference panel. We then sought to evaluate the genetic effects of our lead results on adult measured traits by using findings from previously conducted GWAS in independent adult cohorts (Supplementary Table 2, *Underlying data,* O Nunain *et al.*, 2021a). These were the studies by ( Kettunen *et al.*, 2016; Locke *et al.*, 2015; Richardson *et al.*, 2020c; Willer *et al.*, 2013). If the exact SNP was not present in these results, we used a proxy SNP based on r ^2^ > 0.8 using the same reference panel as before.

***Gene set and functional analysis using tissue-specific and pathway datasets.*** We next evaluated whether findings from our GWAS in ALSPAC were enriched for functional tissue types and biological pathways. In doing so, we aimed to recapitulate findings from previous large-scale GWAS, in terms of the responsible tissue types and pathways which play a role in adiposity and lipid synthesis.

This was undertaken by running our results through the Functional Mapping and Annotation ( FUMA) of GWAS bioinformatic tool ( Watanabe *et al.*, 2017). FUMA was used to assess evidence of enrichment for differentially expressed gene sets using tissue-specific data from the GTEx consortium (v7) ( GTEx Consortium *et al.*, 2017), and evaluate overrepresentations of associated genes on established biological pathways using data from the Reactome database ( Fabregat *et al.*, 2017). We also used the Multi-marker Analysis of GenoMic Annotation (MAGMA) ( de Leeuw *et al.*, 2015) approach to investigate associations between gene sets and each GWAS trait. This was to elucidate potentially overlooked association signals using single SNP analyses in the GWAS.

***Genetic correlations with later life cardiometabolic disease.*** LD score regression was then undertaken to investigate the genetic correlation between our GWAS of early life risk factors and later life cardiometabolic outcomes ( Bulik-Sullivan *et al.*, 2015b). These were coronary artery disease (CAD) ( Nikpay *et al.*, 2015), type 2 diabetes (T2D) ( Mahajan *et al.*, 2018), hypertension and hypercholesterolemia ( Elsworth *et al.*, 2020). LD score regression was conducted using LDSC software ( Bulik-Sullivan *et al.*, 2015a). The **χ** ^2 ^values were calculated for each early life trait, and we only undertook LD score regression for exposures with a coefficient of 1.02 or higher. These guidelines are provided by the authors of this method, as they suggest that traits with values lower than this threshold may yield unreliable results ( Bulik-Sullivan *et al.*, 2015a).

## Results

### Genome-wide association studies of childhood adiposity and lipoprotein lipids

**Figure 1.**
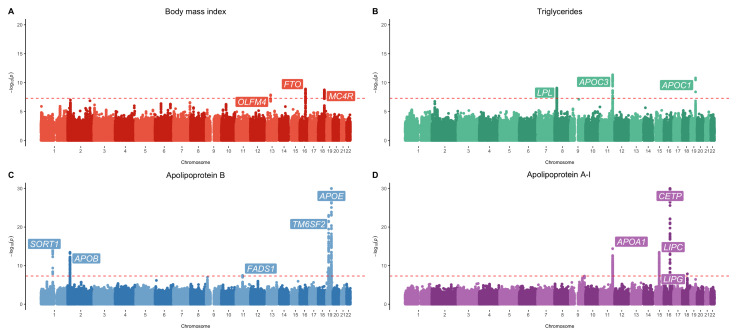
Manhattan plots for body mass index, triglycerides, apolipoprotein B and apolipoprotein A-I. Manhattan plots for genome-wide association studies of early life measures of **A**) body mass index, **B**) triglycerides, **C**) apolipoprotein B and **D**) apolipoprotein A-I. The red dashed line indicates the conventional genome-wide correction threshold of P < 5×10 ^-8^.

Our GWAS analyses identified 14 unique loci associated with at least one measure of early life adiposity based on conventional genome-wide corrections (P<5×10 ^-8^, Table 1). Repeating GWAS analyses with further adjustment for the top 10 principal components identified very little differences in the effect estimates for our top hits, with all their corresponding p-values remaining robust to P<5×10 ^-8^ (Supplementary Table 3, *Underlying data,* O Nunain *et al.*, 2021a). Manhattan plots illustrating results for a selection of the cardiometabolic exposures analysed (BMI, triglycerides, apolipoprotein B and apolipoprotein A-I) can be found in Figure 1.

**Table 1.** Genome-wide association study results for measures of childhood adiposity. A summary of the genetic loci identified in the genome-wide association studies which reached the conventional p-value threshold of 5×10 ^-8^. CHR - Chromosome, BP - base position, SE - standard error, P - p-value.

Trait	Lead SNP	CHR	BP	Gene	Effect allele	Other allele	Beta	SE	P
Apolipoprotein A-I	rs613808	11	116710968	*APOA1*	A	G	0.196064	0.0248607	4.02E-15
Apolipoprotein A-I	rs2070895	15	58723939	*LIPC*	A	G	0.207788	0.0273232	3.54E-14
Apolipoprotein A-I	rs17231506	16	56994528	*CETP*	T	C	0.289439	0.0229323	7.96E-36
Apolipoprotein A-I	rs77960347	18	47109955	*LIPG*	G	A	0.578242	0.101669	1.38E-08
Apolipoprotein B	rs7528419	1	109817192	*SORT1*	G	A	-0.209157	0.0267949	7.51E-15
Apolipoprotein B	rs580889	2	21290067	*APOB*	C	T	-0.216929	0.0285166	3.48E-14
Apolipoprotein B	rs174548	11	61571348	*FADS1*	G	C	-0.131635	0.0237988	3.39E-08
Apolipoprotein B	rs8107974	19	19388500	*TM6SF2*	T	A	-0.407674	0.0402857	8.79E-24
Apolipoprotein B	rs7412	19	45412079	*APOE*	T	C	-0.796266	0.039398	1.99E-86
Body mass Index	rs4477562	13	54104968	*OLFM4*	T	C	0.447695	0.0786661	1.33E-08
Body mass Index	rs55872725	16	53809123	*FTO*	T	C	0.321356	0.0528212	1.25E-09
Body mass Index	rs6567160	18	57829135	*MC4R*	C	T	0.376185	0.0624368	1.80E-09
HDL cholesterol	rs7946869	11	116963312	*APOA1*	T	C	0.162588	0.0289905	2.18E-08
HDL cholesterol	rs1077835	15	58723426	*LIPC*	G	A	0.192653	0.027557	3.19E-12
HDL cholesterol	rs17231506	16	56994528	*CETP*	T	C	0.40379	0.0227858	1.19E-67
HDL cholesterol	rs6857	19	45392254	*APOE*	T	C	-0.171339	0.0308595	3.01E-08
LDL cholesterol	rs599839	1	109822166	*SORT1*	G	A	-0.192309	0.0269997	1.26E-12
LDL cholesterol	rs580889	2	21290067	*APOB*	C	T	-0.199764	0.02869	3.89E-12
LDL cholesterol	rs174548	11	61571348	*FADS1*	G	C	-0.149623	0.0238879	4.16E-10
LDL cholesterol	rs58542926	19	19379549	*TM6SF2*	T	C	-0.389789	0.0410813	3.94E-21
LDL cholesterol	rs7412	19	45412079	*APOE*	T	C	-0.718021	0.0400997	6.04E-69
Triglycerides	rs11984636	8	19885726	*LPL*	C	T	-0.221942	0.0361091	8.71E-10
Triglycerides	rs2072560	11	116661826	*APOC3*	T	C	0.332775	0.0479112	4.39E-12
Triglycerides	rs584007	19	45416478	*APOC1*	A	G	-0.159748	0.0236322	1.59E-11

Results from this analysis included well established loci known to influence cardiometabolic traits in adulthood, such as *FTO* (P=1.25×10 ^-9^) and *MC4R* (P=1.80x10 ^-9^) associated with BMI, *CETP* (P=1.19×10 ^-67^) associated with HDL cholesterol, *SORT1* (P=1.26×10 ^-12^) and *FADS1* (P=4.16×10 ^-10^) associated with LDL cholesterol, *APOA1* (P=4.02×10 ^-15^) associated with apolipoprotein A-I, *APOB* (P=3.48×10 ^-14^) associated with apolipoprotein B, *LPL* (P=8.71×10 ^-10^) and *APOC3* (P=4.39×10 ^-12^) associated with triglycerides and various other known lipid loci (including *LIPC*, *LIPG* and *APOE*) *.* All the loci have also been identified previously in independent adult cohorts (Supplementary Table 4, *Underlying data,* O Nunain *et al.*, 2021a), suggesting that these loci begin to strongly exert their effects on adiposity and lipids traits in early life.

### Functional enrichment and pathway analysis

**Figure 2.**
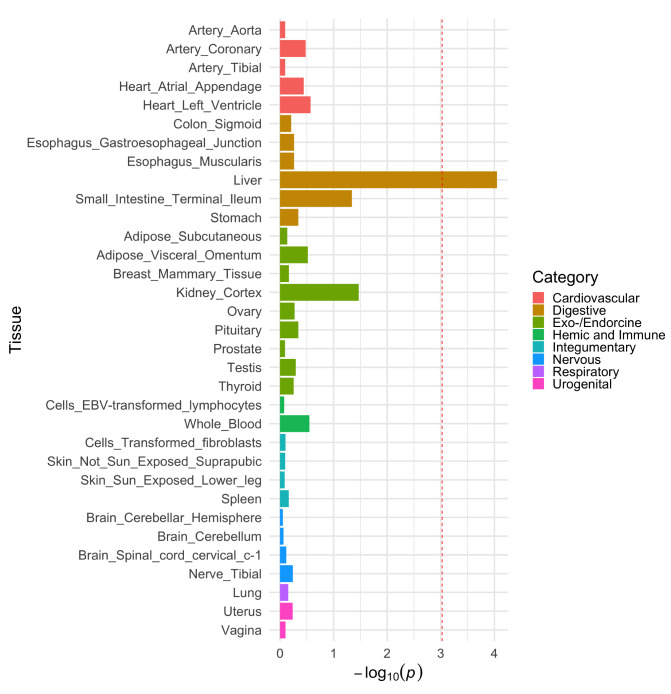
Tissue enrichment for apolipoprotein A-I. Evidence of enrichment for genetic variants robustly associated with apolipoprotein A-I levels in childhood and tissue-specific gene expression derived from human samples. The red dotted line represents the multiple testing adjusted P value threshold.

Leveraging tissue-specific data from the GTEx consortium (v7) ( GTEx Consortium *et al.*, 2017) using the FUMA platform ( Watanabe *et al.*, 2017) suggested that the genes underlying our GWAS hits for lipoprotein lipids are expressed predominantly in liver tissue (Supplementary Figures 1–4, *Extended data,* O Nunain *et al.*, 2021b), as it was the most enriched tissue type for HDL cholesterol, triglycerides and apolipoprotein B. Liver tissue also provided the strongest evidence of enrichment for apolipoprotein A-I as depicted in Figure 2. Undertaking gene-set enrichment analyses using data from the Reactome database ( Fabregat *et al.*, 2017) suggested that these genes were overrepresented on pathways involved in the metabolism and transport of lipids and lipoproteins (Supplementary Figures 5–9, *Extended data,* O Nunain *et al.*, 2021b). We were unable to run enrichment analyses for the BMI GWAS results due to only one SNP being reliably mapped to a gene by FUMA.

Conducting gene-based tests on our results using MAGMA (Multi-marker Analysis of GenoMic Annotation) ( de Leeuw *et al.*, 2015) identified evidence of association for additional genetic loci which did not meet GWAS corrections in the initial analysis (Supplementary Figures 10–15, *Extended data,* O Nunain *et al.*, 2021b). These included *ADCY3* which was associated with BMI, which encodes adenylate cyclase 3 and has been previously implicated in obesity risk through loss-of-function studies ( Grarup *et al.*, 2018). Likewise, we identified evidence that *HMGCR*, the therapeutic target for statin inhibitors, was associated with LDL cholesterol in our dataset based on age 10 individuals from the ALSPAC cohort.

### Assessing genome-wide genetic correlations between childhood adiposity and lipids with later life cardiometabolic disease

BMI, SBP, triglycerides and apolipoprotein B provided **χ** ^2^ values > 1.02 and were eligible for genetic correlation analyses (Supplementary Table 5, *Underlying data,* O Nunain *et al.*, 2021a). Applying LD score regression suggested that our results for childhood BMI were genetically correlated with later life CAD (rG=0.19, 95% CI=0.03 to 0.35, P=0.02), T2D (rG=0.35, 95% CI=0.18 to 0.51, P=3.28x10 ^-5^) and hypertension (rG=0.20, 95% CI=0.07 to 0.32, P=0.002). Similar results were found for childhood SBP; CAD (rG=0.26, 95% CI=0.07 to 0.46, P=0.009), T2D (rG=0.30, 95% CI=0.15 to 0.45, P=1.00×10 ^-4^) and hypertension (rG=0.37, 95% CI=0.19 to 0.55, P=6.57×10 ^-5^).

**Figure 3.**
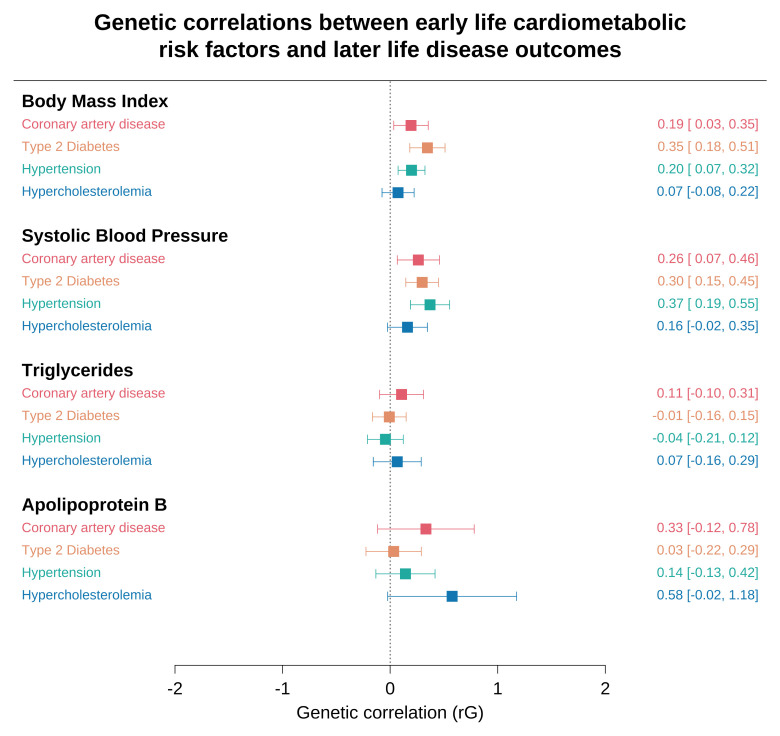
Genetic correlations between early life cardiometabolic risk factors and later life disease outcomes. Forest plots for the linkage disequilibrium (LD) score regression results between early life cardiometabolic risk factors and later life disease outcomes. Genetic correlation coefficients and confidence intervals are shown on the right-hand side. Diastolic blood pressure, high density lipoprotein cholesterol, low density lipoprotein cholesterol and apolipoprotein A-I were not included in this analysis due to having a mean *χ* ^2 ^< 1.02 suggesting that their correlations may be unreliable.

There was weak evidence of a genetic correlation between childhood triglycerides and apolipoprotein B with later-life disease outcomes (Supplementary Table 6, *Underlying data,* O Nunain *et al.*, 2021a). In particular, the wide confidence intervals for apolipoprotein B is likely attributed to the sample size of our GWAS. As such, there were central correlation estimates, which despite being high (e.g. rG=0.58 for hypercholesterolemia), lacked the precision to conclude strong evidence of a genetic correlation. A forest plot of all results from LD score regression analyses can be found in Figure 3.

## Discussion

In this study we provide evidence that there are genetic variants associated with adiposity and lipoprotein lipids which begin to exert their effects as early as age 10 in the life course. The variants robustly associated with lipoprotein lipid traits were enriched for genetic loci whose genes are predominantly expressed in liver tissue and overrepresented on lipid synthesis pathways, supporting their validity as genuine biological effects. Furthermore, we identified strong evidence of genetic correlations between childhood BMI and SBP with later life cardiometabolic disease outcomes.

Our genome-wide association study in a population of young individuals suggested that genetic variation at 14 unique loci has an influence on adiposity and dyslipidaemia even before reaching puberty. Amongst our hits were well-known cardiometabolic loci previously identified in cohorts of adults, such as *FTO* (P=1.25×10 ^-9^ with BMI), *MC4R* (P=1.80×10 ^-9^ with BMI), *LPL* (P=8.71×10 ^-10^ with triglycerides), *CETP* (P=1.19×10 ^-67^ with HDL cholesterol) and *SORT1* (P=1.26×10 ^-12^ with LDL cholesterol). Moreover, the association signals at the *APOA1* locus with apolipoprotein A-I (P=4.02×10 ^-15^) and the *APOB* locus with apolipoprotein B (P=3.48×10 ^-14^) are very likely real biological effects given that they reside at the coding genes responsible for these lipid-related proteins ( Zannis *et al.,* 2001). The early influence of *APOB* on apolipoprotein B levels is of particular interest from a cardiovascular disease prevention perspective, given that there is increasing evidence highlighting the crucial role it plays in coronary heart disease risk ( Holmes & Ala-Korpela, 2019; Richardson *et al.*, 2020c).

Additional downstream analysis and evaluation of our GWAS results provided evidence to reinforce that the genetic variants begin to exert their effects very early in the life course. For instance, using tissue-specific gene expression data from the GTEx consortium suggested that the genes underlying our GWAS hits for lipid-related traits are predominantly expressed in liver tissue. This fits with the biology concerning these traits, given that many lipid and lipoproteins are hepatically synthesised ( Dietschy *et al.*, 1993). Moreover, we identified evidence of an overrepresentation of these genes on curated biological pathways concerning lipid metabolism and transport.

Gene-based analyses provided evidence of additional genes not identified in the single SNP GWAS. These included *ADCY3*, which was associated with BMI and previously shown through loss-of-function analyses to influence obesity and T2D risk ( Grarup *et al.*, 2018). There was also evidence of association between genetic variation at *HMGCR* and LDL cholesterol, which is well-established given that the protein product of this gene (HMG-CoA reductase) is pharmacologically inhibited by statin therapy to lower cholesterol levels ( Cholesterol Treatment Trialists’ (CTT) Collaboration *et al.*, 2010). These findings highlight how early in the life course these genetic effects begin to influence weight and lipid-related traits, suggesting that a long window of opportunity exists to lower cardiovascular disease risk through lifestyle modifications.

To our knowledge, no previous studies have investigated the genetic correlation between childhood blood pressure and lipoprotein lipids with cardiometabolic disease in adulthood. Despite our GWAS sample sizes being modest, we found evidence for a genetic overlap between childhood SBP with coronary heart disease and hypertension in later life. Furthermore, there was strong evidence of a genetic correlation between childhood BMI and T2D, a result that supports recent findings ( Tekola-Ayele *et al.*, 2019; Vogelezang *et al.*, 2020). The genetic correlation between childhood SBP and T2D we identified may be attributed to the vertical pleiotropy which exists between BMI and SBP (i.e. high BMI raising blood pressure levels) ( Wade *et al.*, 2018).

A shared genetic basis may partially explain the association between childhood BMI and later life cardiometabolic disease seen in observational studies ( Reilly & Kelly, 2011). However, given recent evidence, it is likely that childhood adiposity influences adulthood disease risk due to its persistent effect throughout the life course ( Juonala *et al.*, 2011). Although Mendelian randomization studies have been undertaken to support this for childhood adiposity ( Richardson *et al.*, 2020b; Richardson *et al.*, 2020a), future research is required to investigate the direct and indirect effects of childhood blood pressure and lipoprotein lipid traits on later life disease risk. Sufficiently powered sample sizes for these traits in the future will likely facilitate such endeavours, allowing a large number of robustly associated genetic variants to be used as instrumental variables.

In terms of study limitations, the relatively modest sample size of our childhood GWAS (in comparison to modern standards) limited the statistical power of our study, and hence our ability to detect associations. It is likely this is the reason we didn’t observe any SNP associations for SBP after adjusting for conventional multiple-testing corrections applied in GWAS (i.e. P<5×10 ^-8^), although a recent high-powered GWAS, of which ALSPAC was a participating study, identified two SNPs associated with SBP in childhood ( Parmar *et al.*, 2016). Furthermore, the modest sample size of the GWAS also limited the power of our downstream analyses, particularly the LD score regression which is indicated by the low *χ* ^2^ values of several traits.

In conclusion, our findings suggest that future GWAS endeavours should focus on traits during childhood to elucidate variants which have lifelong effects. These will also pave the way for Mendelian randomization analyses to disentangle the contribution of early life exposures to disease risk, independent of the same exposures measured in adulthood. Doing so can help discern whether genetic correlations between childhood traits and disease outcomes, such as those identified in our study, are due to either a direct or indirect effect of early-life risk factors.

## Data availability

### Underlying data

ALSPAC data access is through a system of managed open access. The steps below highlight how to apply for access to the data included in this article, and all other ALSPAC data:

- Please read the ALSPAC access policy which describes the process of accessing the data and samples in detail, and outlines the costs associated with doing so.
- You may also find it useful to browse our fully searchable research proposals database, which lists all research projects that have been approved since April 2011.
- Please submit your research proposal for consideration by the ALSPAC Executive Committee. You will receive a response within 10 working days to advise you whether your proposal has been approved.

Figshare: Supplementary tables for a genome-wide association study of childhood adiposity and blood lipids, https://doi.org/10.6084/m9.figshare.15134409.v3 ( O Nunain *et al.*, 2021a)

This project contains the following underlying data:

- Supplementary Table 1: Trait characteristics from the ALSPAC cohort at mean age 9.9
- Supplementary Table 2: Dataset of adult populations used in this study to evaluate genetic effects identified in ALSPAC
- Supplementary Table 3: Genome-wide association study results for measures of childhood adiposity adjusted for population stratification
- Supplementary Table 4: Evaluation of genome-wide association study hits in adult populations
- Supplementary Table 5: χ2 coefficients for each childhood exposure to assess eligiblity for genetic correlation analyses
- Supplementary Table 6: Linkage disequilibrium score regression results

### Extended data

Figshare: Extended data for a genome-wide association study of childhood adiposity and blood lipids, https://doi.org/10.6084/m9.figshare.15172824.v3 ( O Nunain *et al*., 2021b)

This project contains the following:

- Supplementary figures for a genome-wide association study of childhood adiposity and blood lipids
