# Supplementary material for: A genome-wide association study of childhood adiposity and blood lipids
Source: Wellcome Open Res. 2023 Mar 23;6:303. Originally published 2021 Nov 10. [Version 2] doi: 10.12688/wellcomeopenres.16928.2 (PMC11411246; doi:10.12688/wellcomeopenres.16928.2)
Supplement: Peer review history

# Reviewer report: Reviewer response for version 2

**DOI:** 10.21956/wellcomeopenres.21307.r105991

**Published:** 2024-11-02

**Authors:** Sheridan H. Littleton

**Affiliations:** 1 Department of Clinical Biochemistry, University of Cambridge, Cambridge, England, UK

In this article by O'Nunain et al., the authors describe a collection of genome-wide association studies for measures of adiposity and lipids from children in the ALSPAC cohort. They report loci significantly associated with Apolipoprotein A-I, Apolipoprotein B, BMI, HDL, LDL, and triglycerides. As expected, they found some of the most robust known associations such as FTO and MC4R. Interestingly, all of the identified loci have been independently identified in adult cohorts, suggesting that these loci begin to effect phenotypic variation early in life. They then went on to perform functional enrichment, pathway analyses, and LDSR, identifying that genes at lipoprotein lipid loci were enriched in liver tissue and there are correlations between childhood disease risk and disease outcomes in later life. 

 I believe this study is clear and provides sufficient detail to understand and replicate their findings. The authors recognize that they have a smaller sample size compared to other studies and discuss this as a limitation. The novelty of this study comes from the comparison between childhood blood lipids and cardiometabolic traits in adulthood. There is a need to characterize genetic variation that increases disease risk early on in life, influencing future diagnostic and treatment methods that could improve the lives of children and future adults. This work is a direct contribution to the effort of identifying important early-life risk factors.

Is the work clearly and accurately presented and does it cite the current literature?

Yes

If applicable, is the statistical analysis and its interpretation appropriate?

I cannot comment. A qualified statistician is required.

Are all the source data underlying the results available to ensure full reproducibility?

Yes

Is the study design appropriate and is the work technically sound?

Yes

Are the conclusions drawn adequately supported by the results?

Yes

Are sufficient details of methods and analysis provided to allow replication by others?

Yes

Reviewer Expertise:

Genetics of childhood obesity, genome-wide association study functional follow-up

I confirm that I have read this submission and believe that I have an appropriate level of expertise to confirm that it is of an acceptable scientific standard.

# Reviewer report: Reviewer response for version 2

**DOI:** 10.21956/wellcomeopenres.21307.r109001

**Published:** 2024-11-01

**Authors:** Matthew C. Pahl

**Affiliations:** 1 The Children's Hospital of Philadelphia, Philadelphia, Pennsylvania, USA

To investigate the relationship between multiple relevant traits for childhood obesity and subsequent cardiovascular/metabolic outcomes, O’Nunain et al. conducted a GWAS examining BMI and several obesity relevant phenotypes, using approximately 5,000 donors from the ALSPAC cohort. Following this, the authors performed genetic correlation analyses between these childhood traits and adult cardiovascular/metabolic phenotypes. The authors have made their summary statistics publicly available on the GWAS catalog.

 While the authors did not identify novel loci, their efforts successfully replicated major known loci from previous studies in adults and children, which was expected given the modest sample size used. Several larger studies have examined BMI in children, but fewer have assessed the genetics of other obesity related phenotypes in childhood. Demonstrating that the genetic signals found in adults are detectable in a young cohort is both novel and beneficial to the field. The study employed standard methodology, with results described and presented clearly. However, the paper could be strengthened by a more in-depth discussion of their findings in the context of previous research and clarifying some details on their methods.

**Major Points:**

 The FUMA analysis for apolipoprotein B and LDL cholesterol shown in Supplementary Figure 1 also appears to detect enrichment in several brain-related tissue annotations, in addition to liver enrichment reported in the text. Was this result expected given existing literature?

 Given that the authors discuss their findings in the context of when the genetic architecture for obesity-relevant traits is established (Discussion, first sentence), a more thorough discussion of the existing literature on this topic is warranted. Helgeland et al. (2022) previously conducted a GWAS of BMI across multiple childhood time points (from birth to 10 years, extended to 18 years using ALSPAC data). Many classic BMI loci (e.g., FTO, MC4R) were found to be significantly associated only from approximately age 7. These results, along with other relevant findings, should be discussed with the present study's results.

 For completeness, the authors should provide additional details about the LD score regression analysis. Specifically, did they use the 1000 Genomes Project as the LD reference? Was the HapMap3 SNP set used as the reference? Were these analyses conducted using hg19 or hg38 references?  Also please provide more detail on the Polygenic risk score analyses.

 Several supplementary Tables were not available on Figshare (only six files were accessible). I believe the table with the Polygenic risk score analyses were missing.

Is the work clearly and accurately presented and does it cite the current literature?

Yes

If applicable, is the statistical analysis and its interpretation appropriate?

Yes

Are all the source data underlying the results available to ensure full reproducibility?

Partly

Is the study design appropriate and is the work technically sound?

Yes

Are the conclusions drawn adequately supported by the results?

Yes

Are sufficient details of methods and analysis provided to allow replication by others?

Partly

Reviewer Expertise:

Population genetics, Variant annotation, Genomics, Metabolic Disease, Gene Regulation, Epigenetics

I confirm that I have read this submission and believe that I have an appropriate level of expertise to confirm that it is of an acceptable scientific standard, however I have significant reservations, as outlined above.

# Reviewer report: Reviewer response for version 2

**DOI:** 10.21956/wellcomeopenres.21307.r92540

**Published:** 2024-09-18

**Authors:** Xinghao Yu, Huimin Lu

**Affiliations:** 1 Soochow University, Suzhou, Jiangsu, China; 2 First affiliated hospital of Soochow university, Soochow University, Suzhou, Jiangsu, China

This is a interesting topic exploring the genetic determinants of child adiposity and blood lipids. However, I have several concerns:

1. It appears that Table S7-S8 is missing from the appendix; please check and ensure these are included.
2. While childhood obesity and hyperlipidemia are indeed related to cardiovascular disease, the focus should not be solely on high BMI. In fact, low BMI is also associated with elevated cardiovascular risk. The authors have only evaluated the linear relationship between BMI and disease risk, which may not be the most appropriate approach. Given the availability of individual-level GWAS data, I recommend assessing the nonlinear associations between the variables of interest and the risk of later-life diseases, considering both phenotypic and genetic aspects.
3. The authors mention constructing GRS scores to evaluate the genetic correlation of eight cardiometabolic traits measured during childhood and adulthood. However, there are missing details in the methodology, and it's unclear whether the authors assessed the correlation between GRS scores and the phenotypic traits of cardiometabolic outcomes, or the correlation with GRS scores of adult cardiometabolic traits. These are two distinct strategies, and I would categorize the former as a single-sample MR analysis.
4. It seems that the risk loci identified in this study have already been observed in independent adult cohorts. Given this, what are the practical implications of this study? Do the adult cohort results suggest the need for early intervention? Or could it imply that factors such as obesity tend to follow a linear trajectory across the life course?

Is the work clearly and accurately presented and does it cite the current literature?

Yes

If applicable, is the statistical analysis and its interpretation appropriate?

Partly

Are all the source data underlying the results available to ensure full reproducibility?

No

Is the study design appropriate and is the work technically sound?

Yes

Are the conclusions drawn adequately supported by the results?

Partly

Are sufficient details of methods and analysis provided to allow replication by others?

Partly

Reviewer Expertise:

Genetic statistics

We confirm that we have read this submission and believe that we have an appropriate level of expertise to confirm that it is of an acceptable scientific standard, however we have significant reservations, as outlined above.

# Reviewer report: Reviewer response for version 2

**DOI:** 10.21956/wellcomeopenres.21307.r55706

**Published:** 2023-04-11

**Authors:** Veera Rajagopal

**Affiliations:** 1 Department of Biomedicine, Aarhus University, Aarhus, Denmark

The authors have sufficiently addressed my comments, and I have no further comments.

Is the work clearly and accurately presented and does it cite the current literature?

Yes

If applicable, is the statistical analysis and its interpretation appropriate?

Partly

Are all the source data underlying the results available to ensure full reproducibility?

No

Is the study design appropriate and is the work technically sound?

Yes

Are the conclusions drawn adequately supported by the results?

Partly

Are sufficient details of methods and analysis provided to allow replication by others?

Yes

Reviewer Expertise:

GWAS, statistical genetics and psychiatric genetics

I confirm that I have read this submission and believe that I have an appropriate level of expertise to confirm that it is of an acceptable scientific standard.

# Reviewer report: Reviewer response for version 1

**DOI:** 10.21956/wellcomeopenres.18679.r47034

**Published:** 2022-01-24

**Authors:** Samuel Jones

**Affiliations:** 1 Institute for Molecular Medicine (FIMM), HiLIFE, University of Helsinki, Helsinki, Finland

The authors report on a series of eight GWAS of adipose- and lipid-related traits (BMI, triglycerides, LDL- and HDL-cholesterol, Apolipoproteins A-I and B, and systolic and diastolic blood pressure) in a cohort of approximately 5,000 participants of European ancestry. Despite the limited sample size, the authors report the identification of 24 genome-wide significant genetic associations in 14 unique loci across their eight phenotypes. Amongst the identified loci were those previously associated in later-life GWAS for the equivalent traits, such as FTO and MC4R (BMI), APOA1, and APOB (apolipoproteins A-I and B, respectively) and others. The authors follow up their findings by firstly interrogating the associations through genetic correlation with later-life cardiometabolic phenotypes, finding moderate but significant genetic overlap between early-life systolic blood pressure and later-life CAD and hypertension and early-life BMI with later-life type 2 diabetes, though report low heritability estimates for the early-life phenotypes. This was followed by gene-set enrichment analysis in an attempt to understand the biological mechanisms in which the identified genetic variants were implicated, with genes involved in metabolism and lipid transport pathways and those expressed in liver tissue showing evidence of being enriched. The authors conclude that their results demonstrate the ability to detect the early effect of genetic factors on adipose and lipid traits and that further work should be undertaken to understand the effects of (more acute) early-life exposure versus the cumulative (chronic) effect of life-long exposure to these genetic factors.

 I feel this manuscript is an important addition to the literature on early-life traits and am pleased to see that focus is not just on trying to replicate findings from later-life GWAS for the equivalent phenotypes. That being said, more could be done to help the reader understand whether the genetics of these early-life phenotypes really are distinct from the genetics identified in later-life GWAS. I have a few suggestions and questions that I feel need to be addressed before the manuscript should be accepted.

Major Comments

- In the results and discussion sections, it is mentioned that well-known loci were seen, but did the variants identified represent the same signal as in the later-life GWAS? If the variants aren’t the same, what is the LD between your variant and the previously reported one? I’m not sure if you can qualify your discussion of overlapping signals unless we know whether the lead variants are in LD.
- An obvious question is: “How genetically correlated are early-life phenotypes with later-life phenotypes?”. I understand that the early-life heritability estimates are low, given the small sample sizes, but it would help contextualise the genetic correlations with later-life cardiometabolic phenotypes that you do report.
- The discussion mentions that the signals at the APOA1 and APOB loci are “very likely real biological effects given that they reside at the coding genes...”, but what are the functions of the lead variants at these loci? Are they coding variants within the genes or are they within known eQTLs for these genes? If so, this is definitely worth including in the results/discussion. If not, I don’t know if you can claim that they are “likely real biological effects”, unless there is other evidence to link these variants to the specific genes.
- Is there a reason that 1) related samples were removed and 2) genotypes were converted to best-guess for GWA analysis? In an ideal situation, I would recommend rerunning the GWAS software that handles related samples and imputed data – is this a possibility?
- It is good practice to make GWAS summary statistics available for use by the wider scientific community, but in your Data Availability section, I don’t see any mention of accessing these. Are you planning to make these available? If so, please make it clear how to access them. If not, what are the justifications for not making these available?

Minor Comments

- Would it be possible to add the N of the largest GWAS to the methods section of the abstract, to give the reader a better idea of the cohort size without having to delve into the manuscript?
- If there is space, perhaps add a sentence in the background section of the abstract on why elucidating the genetics is important for these traits?
- In Table 1 and Supp Tables 3 and 4, can you make it clear which genome build the positions are in? This is incredibly useful when other researchers come to use your published results.
- Could you clarify how the genes were identified for each locus? Were they the nearest genes? Or are these the genes mapped using FUMA GWAS?
- Would it be possible to highlight whether the lead variants identified are intergenic, intronic, exonic, etc.?
- In the limitations, where the study that identified two SNPs associated with childhood SBP is mentioned, can you add the sample size of that study to provide some context to their findings in relation to yours? Did you see even nominal associations for these reported variants in your results? Please report negative findings too!

Is the work clearly and accurately presented and does it cite the current literature?

Yes

If applicable, is the statistical analysis and its interpretation appropriate?

Yes

Are all the source data underlying the results available to ensure full reproducibility?

Partly

Is the study design appropriate and is the work technically sound?

Partly

Are the conclusions drawn adequately supported by the results?

Partly

Are sufficient details of methods and analysis provided to allow replication by others?

Yes

Reviewer Expertise:

Statistical genetics, genetic epidemiology, population genetics

I confirm that I have read this submission and believe that I have an appropriate level of expertise to confirm that it is of an acceptable scientific standard, however I have significant reservations, as outlined above.

# Response

**Published:** 2023-03-11

**Authors:** Tom Richardson

**Affiliations:** MRC Integrative Epidemiology Unit, UK

**Reviewer #2** The authors report on a series of eight GWAS of adipose- and lipid-related traits (BMI, triglycerides, LDL- and HDL-cholesterol, Apolipoproteins A-I and B, and systolic and diastolic blood pressure) in a cohort of approximately 5,000 participants of European ancestry. Despite the limited sample size, the authors report the identification of 24 genome-wide significant genetic associations in 14 unique loci across their eight phenotypes. Amongst the identified loci were those previously associated in later-life GWAS for the equivalent traits, such as FTO and MC4R (BMI), APOA1, and APOB (apolipoproteins A-I and B, respectively) and others. The authors follow up their findings by firstly interrogating the associations through genetic correlation with later-life cardiometabolic phenotypes, finding moderate but significant genetic overlap between early-life systolic blood pressure and later-life CAD and hypertension and early-life BMI with later-life type 2 diabetes, though report low heritability estimates for the early-life phenotypes. This was followed by gene-set enrichment analysis in an attempt to understand the biological mechanisms in which the identified genetic variants were implicated, with genes involved in metabolism and lipid transport pathways and those expressed in liver tissue showing evidence of being enriched. The authors conclude that their results demonstrate the ability to detect the early effect of genetic factors on adipose and lipid traits and that further work should be undertaken to understand the effects of (more acute) early-life exposure versus the cumulative (chronic) effect of life-long exposure to these genetic factors. I feel this manuscript is an important addition to the literature on early-life traits and am pleased to see that focus is not just on trying to replicate findings from later-life GWAS for the equivalent phenotypes. That being said, more could be done to help the reader understand whether the genetics of these early-life phenotypes really are distinct from the genetics identified in later-life GWAS. I have a few suggestions and questions that I feel need to be addressed before the manuscript should be accepted. **Major Comments**

- In the results and discussion sections, it is mentioned that well-known loci were seen, but did the variants identified represent the same signal as in the later-life GWAS? If the variants aren’t the same, what is the LD between your variant and the previously reported one? I’m not sure if you can qualify your discussion of overlapping signals unless we know whether the lead variants are in LD.

**To clarify, evaluations of the effect estimates in later-life GWAS reported in Table S4 are the exact same variant as the ones found to be lead markers in ALSPAC analyses. Therefore, LD is not an issue for these look-ups. This has been clarified on page 8.** **“All the loci have also been identified previously in independent adult cohorts (effect estimates for lead variants found Supplementary Table 4, Underlying data, O Nunain et al., 2021a), suggesting that these loci begin to strongly exert their effects on adiposity and lipids traits in early life.”**

- An obvious question is: “How genetically correlated are early-life phenotypes with later-life phenotypes?”. I understand that the early-life heritability estimates are low, given the small sample sizes, but it would help contextualise the genetic correlations with later-life cardiometabolic phenotypes that you do report.

**As suggested by reviewer #1, we have conducted a polygenic risk score analysis to evaluate genetic correlations between childhood and adulthood phenotypes. This is now reported on page 8:** **“Additionally, generating whole genome polygenic risk scores in the UKB using estimates derived from ALSPAC analyses found strong evidence of association for all 8 traits (Supplementary Table 5, Underlying data O Nunain et al., 2021a), suggesting a high level of genetic correlation between their measured obtained during childhood and adulthood.”**

- The discussion mentions that the signals at the APOA1 and APOB loci are “very likely real biological effects given that they reside at the coding genes...”, but what are the functions of the lead variants at these loci? Are they coding variants within the genes or are they within known eQTLs for these genes? If so, this is definitely worth including in the results/discussion. If not, I don’t know if you can claim that they are “likely real biological effects”, unless there is other evidence to link these variants to the specific genes.

**We have now added VEP annotations to Table 1 as also recommended by reviewer #1.**

- Is there a reason that 1) related samples were removed and 2) genotypes were converted to best-guess for GWA analysis? In an ideal situation, I would recommend rerunning the GWAS software that handles related samples and imputed data – is this a possibility?

**GWAS data in ALSPAC has been prepared internally by the cohort and provided to researchers in the current format to ensure the reproducibility of results. The changes suggested would therefore require an updated application to ALSPAC. **

- It is good practice to make GWAS summary statistics available for use by the wider scientific community, but in your Data Availability section, I don’t see any mention of accessing these. Are you planning to make these available? If so, please make it clear how to access them. If not, what are the justifications for not making these available?

**Many thanks for this suggestion. We have now uploaded our full summary statistics to the GWAS catalog (accession numbers GCST90104677 to GCST90104684) as mentioned on page 13 of the manuscript:** **“The full set of summary statistics for the 8 GWAS conducted in this study can be found on the GWAS catalog (accession numbers GCST90104677 to GCST90104684”** **Minor Comments**

- Would it be possible to add the N of the largest GWAS to the methods section of the abstract, to give the reader a better idea of the cohort size without having to delve into the manuscript?

**We have added sample sizes to the abstract as requested.**

- If there is space, perhaps add a sentence in the background section of the abstract on why elucidating the genetics is important for these traits?

**We have now added the following sentence to the abstract:** **“** **Previous studies have found that genetic variants inherited at birth can begin to exert their effects on cardiometabolic traits during the early stages of the lifecourse.”**

- In Table 1 and Supp Tables 3 and 4, can you make it clear which genome build the positions are in? This is incredibly useful when other researchers come to use your published results.

**We have now clarified that results are reported on the hg19 build of the human genome as recommended in these tables.**

- Could you clarify how the genes were identified for each locus? Were they the nearest genes? Or are these the genes mapped using FUMA GWAS?

**Genes were mapped at each locus based on previous GWAS published in the literature and functional follow-up studies of these loci.** 

- Would it be possible to highlight whether the lead variants identified are intergenic, intronic, exonic, etc.?

**VEP annotations have now been added to Table 1 to address this point.**

- In the limitations, where the study that identified two SNPs associated with childhood SBP is mentioned, can you add the sample size of that study to provide some context to their findings in relation to yours? Did you see even nominal associations for these reported variants in your results? Please report negative findings too!

**We have added the sample size of this study to the discussion (n=8,423) as well as providing a look up for this SNP in our own study (page 12). Previously we reported that two variants surpassed genome-wide corrections, although only one of these was based on blood pressure measured at puberty (as in our study).** **“A previous GWAS (N = 8,423), of which ALSPAC was a participating study, identified one SNPs associated with SBP at puberty (rs872256, P=8.7x10 ^-9^) (** **Parmar et al., 2016** **), which did not reach genome-wide corrections in ALSPAC alone (P=6.4x10 ^-5^ in this study).”**

# Reviewer report: Reviewer response for version 1

**DOI:** 10.21956/wellcomeopenres.18679.r47032

**Published:** 2021-12-07

**Authors:** Veera Rajagopal

**Affiliations:** 1 Department of Biomedicine, Aarhus University, Aarhus, Denmark

O’Nunai *et al.* has performed genome-wide association studies (GWASs) for eight cardio-metabolic traits—body mass index (BMI), systolic and diastolic blood pressure (SBP and DBP), high-density and low-density lipoprotein cholesterol (HDL and LDL), triglycerides (TGL), apolipoprotein A-1 and B (apo-A1, apo-B)—in ~5000 children from ALSPAC cohort. Although the sample size is smaller by many orders of magnitude compared to other existing GWASs, the study is interesting as it evaluates the genetic influences of these cardio-metabolic traits in children as opposed to most other studies that studied mainly adults (with the exception of studies of childhood BMI). The authors report the results following a conventional style. As expected many of the known suspects (e.g. FTO, MC4R, APOE, etc.) show up beautifully in the GWASs, highlighting their strong genetic effects. Gene set enrichment analysis implicates disease relevant tissues and pathways and genetic correlation analyses suggest genetic variants influencing cardio-metabolic quantitative traits in children are the same that influence the risk for cardio-metabolic diseases in adults.

 Given the major—and perhaps the only—strength of this study is that these phenotypes are measured in children, I’d report the results slightly differently. The main questions, as the authors discuss in the paper, to ask in such a sample are

1. Do the genetic variants that influence cardio-metabolic traits and diseases in adulthood also influence in childhood? (The answer to this question is often yes unless there is a strong biological argument to suggest otherwise)
2.  Do the effect sizes of these risk variants differ between childhood and adulthood?

I am not sure if the current version of the paper answers these questions clearly. I recommend the following revisions to improve the manuscript so that it answers the key questions mentioned above.

**Variant level associations:**

 In the current version, the authors report only loci significant above conventional genome-wide significant threshold (5e-8). However, I’d not consider the current analysis as discovery in nature, given that the sample size is too small and there exist GWASs for these traits in very large sample sizes. Reporting genome-wide hits is okay. But a better way to report variant associations is to first take all the variants that are reported as genome-wide significant in the most recent GWASs of each of the eight traits and evaluate their significance in the current sample. The P value threshold can be set based on the number of variants being evaluated. We’d expect only those variants with higher statistical power will replicate in the current study. That is, those variants with large effect size and rare MAF or with moderate effect size and common MAF. This can be visualised using an allele frequency vs effect size plot. For an idea, please refer to figure 3 from the recent preprint from global biobank meta-analysis initiative (Zhou *et al.*, MedRxiv, 2021). ^1^ Reporting such a plot will be very informative and educational for the readers. When replicated and non-replicated variants were differentiated by shape (color differentiating the traits), we would see all the replicated variants falling within the centre zone within a U shape. This kind of visual inspection is important because—firstly, by reproducing the expected pattern it ensures that the analyses were performed properly and secondly, it helps identify outliers that deviate from the expected pattern (e.g. if a variant with sufficient power does not show a significant association) and study them further. Such outliers are the ones that likely have different effects in childhood vs adulthood.

 Additionally, I recommend to compare the effect sizes (standardised betas) of those variants that replicate between childhood and adulthood. Perhaps a scatter plot with effect sizes reported in adult sample GWASs in X axis and effect sizes observed in the current sample in Y axis. Any outliers in this scatter plot might be interesting candidates to study further as they will correspond to variants with differential effects between childhood and adulthood.

**MAGMA gene based analysis and tissue specific enrichment:**

 Gene based analyses and tissue specific enrichment analysis using FUMA do not add anything new and also in such a small sample size I wouldn’t do these analyses. Removing these altogether or reporting them in supplementary will help the readers to focus only on the main findings.

**Genetic correlation analysis:**

 LD score regression based genetic correlation analysis between two traits, say A and B, requires adequate sample sizes for both A and B GWASs. Hence, not an ideal analysis for the GWASs reported in the current paper. An alternative would be to perform a polygenic score analysis and report the betas and P values as we have GWAS for these traits in UK Biobank in huge sample sizes that will serve as training samples and will offer better power to detect genetic associations. It would be more informative if the authors could perform a similar analysis also in a set of adult samples (perhaps a small chunk of UK Biobank sample kept out of the training) and compare the effect sizes between childhood and adulthood. If the polygenic score analysis could not be performed for some reason. I recommend that at least the authors report the LDSC rg for both child and adult GWASs. Otherwise, the genetic correlation analysis results will offer no insight to the readers.

**Minor comments**

1. Please provide the sample size in abstract, methods, results and in the main tables. When you report genome-wide significant variants as a table, it is essential that it also has an N column. It is not fair to expect the readers to go to supplementary tables to learn this crucial piece of information.
2. I recommend the authors to make the full summary statistics publicly available for the readers.

Is the work clearly and accurately presented and does it cite the current literature?

Yes

If applicable, is the statistical analysis and its interpretation appropriate?

Partly

Are all the source data underlying the results available to ensure full reproducibility?

No

Is the study design appropriate and is the work technically sound?

Yes

Are the conclusions drawn adequately supported by the results?

Partly

Are sufficient details of methods and analysis provided to allow replication by others?

Yes

Reviewer Expertise:

GWAS, statistical genetics and psychiatric genetics

I confirm that I have read this submission and believe that I have an appropriate level of expertise to confirm that it is of an acceptable scientific standard, however I have significant reservations, as outlined above.

## References

[1] : Global Biobank Meta-analysis Initiative: powering genetic discovery across human diseases. *medRxiv* .2021; 10.1101/2021.11.19.21266436 10.1101/2021.11.19.21266436 PMC990371636777996

# Response

**Published:** 2023-03-11

**Authors:** Tom Richardson

**Affiliations:** MRC Integrative Epidemiology Unit, UK

O’Nunain *et al.* has performed genome-wide association studies (GWASs) for eight cardio-metabolic traits—body mass index (BMI), systolic and diastolic blood pressure (SBP and DBP), high-density and low-density lipoprotein cholesterol (HDL and LDL), triglycerides (TGL), apolipoprotein A-1 and B (apo-A1, apo-B)—in ~5000 children from ALSPAC cohort. Although the sample size is smaller by many orders of magnitude compared to other existing GWASs, the study is interesting as it evaluates the genetic influences of these cardio-metabolic traits in children as opposed to most other studies that studied mainly adults (with the exception of studies of childhood BMI). The authors report the results following a conventional style. As expected many of the known suspects (e.g. FTO, MC4R, APOE, etc.) show up beautifully in the GWASs, highlighting their strong genetic effects. Gene set enrichment analysis implicates disease relevant tissues and pathways and genetic correlation analyses suggest genetic variants influencing cardio-metabolic quantitative traits in children are the same that influence the risk for cardio-metabolic diseases in adults.  

 Given the major—and perhaps the only—strength of this study is that these phenotypes are measured in children, I’d report the results slightly differently. The main questions, as the authors discuss in the paper, to ask in such a sample are

1. Do the genetic variants that influence cardio-metabolic traits and diseases in adulthood also influence in childhood? (The answer to this question is often yes unless there is a strong biological argument to suggest otherwise)
2. Do the effect sizes of these risk variants differ between childhood and adulthood?

I am not sure if the current version of the paper answers these questions clearly. I recommend the following revisions to improve the manuscript so that it answers the key questions mentioned above.  

**Variant level associations:** In the current version, the authors report only loci significant above conventional genome-wide significant threshold (5e-8). However, I’d not consider the current analysis as discovery in nature, given that the sample size is too small and there exist GWASs for these traits in very large sample sizes. Reporting genome-wide hits is okay. But a better way to report variant associations is to first take all the variants that are reported as genome-wide significant in the most recent GWASs of each of the eight traits and evaluate their significance in the current sample. The P value threshold can be set based on the number of variants being evaluated. We’d expect only those variants with higher statistical power will replicate in the current study. That is, those variants with large effect size and rare MAF or with moderate effect size and common MAF. This can be visualised using an allele frequency vs effect size plot. For an idea, please refer to figure 3 from the recent preprint from global biobank meta-analysis initiative (Zhou *et al.*, MedRxiv, 2021). 1 Reporting such a plot will be very informative and educational for the readers. When replicated and non-replicated variants were differentiated by shape (color differentiating the traits), we would see all the replicated variants falling within the centre zone within a U shape. This kind of visual inspection is important because—firstly, by reproducing the expected pattern it ensures that the analyses were performed properly and secondly, it helps identify outliers that deviate from the expected pattern (e.g. if a variant with sufficient power does not show a significant association) and study them further. Such outliers are the ones that likely have different effects in childhood vs adulthood.

**Many thanks for your suggestion to include an overview of variant level associations to the paper. We have generated the plot you have described to Figure 2 of the manuscript (referenced on page 9).**    

 Additionally, I recommend to compare the effect sizes (standardised betas) of those variants that replicate between childhood and adulthood. Perhaps a scatter plot with effect sizes reported in adult sample GWASs in X axis and effect sizes observed in the current sample in Y axis. Any outliers in this scatter plot might be interesting candidates to study further as they will correspond to variants with differential effects between childhood and adulthood. **We have also added this scatter plot to the manuscript as Supplementary Figure 1 which is referred to on page 8.**    

**Supplementary figure 1: Comparison of variant effect sizes in childhood and adulthood**. Scatter plot depicting the different effect sizes of replicated variants in adulthood and childhood.

**MAGMA gene based analysis and tissue specific enrichment:** Gene based analyses and tissue specific enrichment analysis using FUMA do not add anything new and also in such a small sample size I wouldn’t do these analyses. Removing these altogether or reporting them in supplementary will help the readers to focus only on the main findings.

**MAGMA & FUMA results have been moved to supplementary as recommended.** **Genetic correlation analysis:** LD score regression based genetic correlation analysis between two traits, say A and B, requires adequate sample sizes for both A and B GWASs. Hence, not an ideal analysis for the GWASs reported in the current paper. An alternative would be to perform a polygenic score analysis and report the betas and P values as we have GWAS for these traits in UK Biobank in huge sample sizes that will serve as training samples and will offer better power to detect genetic associations. It would be more informative if the authors could perform a similar analysis also in a set of adult samples (perhaps a small chunk of UK Biobank sample kept out of the training) and compare the effect sizes between childhood and adulthood. If the polygenic score analysis could not be performed for some reason. I recommend that at least the authors report the LDSC rg for both child and adult GWASs. Otherwise, the genetic correlation analysis results will offer no insight to the readers.

**Thank you for this suggestion. We have now conducted polygenic risk score analyses as suggested to evaluate the genetic correlation between our childhood GWAS and measured traits in the UK Biobank (page 8):** **“Additionally, generating whole genome polygenic risk scores in the UKB using estimates derived from ALSPAC analyses found strong evidence of association for all 8 traits (Supplementary Table 5, Underlying data O Nunain *et al.*, 2021a), suggesting a high level of genetic correlation between their measured obtained during childhood and adulthood.”**

**Minor comments**

1. Please provide the sample size in abstract, methods, results and in the main tables. When you report genome-wide significant variants as a table, it is essential that it also has an N column. It is not fair to expect the readers to go to supplementary tables to learn this crucial piece of information.

**Sample sizes for our GWAS have now been added to the sections listed above and Table 1.**

1. I recommend the authors to make the full summary statistics publicly available for the readers.

**We have now uploaded our full summary statistics to the GWAS catalog (accession numbers GCST90104677 to GCST90104684) as mentioned on page 6 of the manuscript:**
